# Perforation of the Large Intestine by a Fish Bone Mimicking Cancer: A Rare Case Report

**DOI:** 10.7759/cureus.85867

**Published:** 2025-06-12

**Authors:** Daniel Herrera Hernández, Estefany Marlen Barragán Jiménez, Pablo Cano Cabrera, Pablo Patricio Flores García, Ivan Yahir Paternina Gonzalez, Diana Laura Páramo Hernández, Ricardo Burciaga Castañeda, Jimena Celeste Treviño Flores

**Affiliations:** 1 Surgery, Hospital General Regional No. 1 Instituto Mexicano del Seguro Social, Tijuana, MEX; 2 Surgery, Hospital General de Zona No. 3, Instituto Mexicano del Seguro Social, Aguascalientes, MEX; 3 Surgery, Hospital Regional de Alta Especialidad ISSSTE, Veracruz, MEX; 4 Surgery, Hospital Universitario Dr. José Eleuterio Gonzalez, Monterrey, MEX; 5 Surgery, Hospital Español de México, Mexico City, MEX; 6 Surgery, Hospital Juárez de México, Mexico City, MEX; 7 Surgery, C.H. ISSSTE Gómez Palacio, Mexico City, MEX

**Keywords:** bowel perforation, fish bone, fish bone perforation, large bowel perforation, surgical acute abdomen

## Abstract

This case report details an 80-year-old male who presented with a 12-day history of progressive left lower quadrant abdominal pain. Imaging studies revealed a mass adjacent to the sigmoid colon with signs suggestive of an abscess, but no foreign body was identified preoperatively. Surgical exploration uncovered a sigmoid colon perforation associated with a sharp foreign object, later confirmed to be a fish bone. Histopathological examination demonstrated an inflammatory response without evidence of malignancy. The patient underwent a segmental colectomy with favorable postoperative recovery. This case underscores the diagnostic challenge of foreign body perforation of the colon, particularly when initial imaging fails to identify the foreign object. It highlights the importance of considering ingested fish bones in the differential diagnosis of colonic perforation in elderly patients, especially those with risk factors such as denture use. The report emphasizes the role of surgical intervention in the diagnosis and management of such rare cases, which can mimic neoplastic processes.

## Introduction

Intestinal perforation caused by a foreign body is exceedingly difficult to diagnose preoperatively and is extremely rare, occurring in less than 1% of cases [1]. In most instances, patients do not recall ingesting the foreign body. Presenting symptoms can closely resemble those of acute appendicitis, diverticulitis, or even generalized peritonitis with signs of perforation, as seen in our patient [2]. Due to intestinal anatomy, the most common site of perforation is the ileum, followed by the rectosigmoid junction [3]. Sometimes, surgical intervention becomes necessary due to the high risk of complications. Below, we describe an unusual case of sigmoid colon perforation caused by a fish bone.

## Case presentation

**Figure 1:**
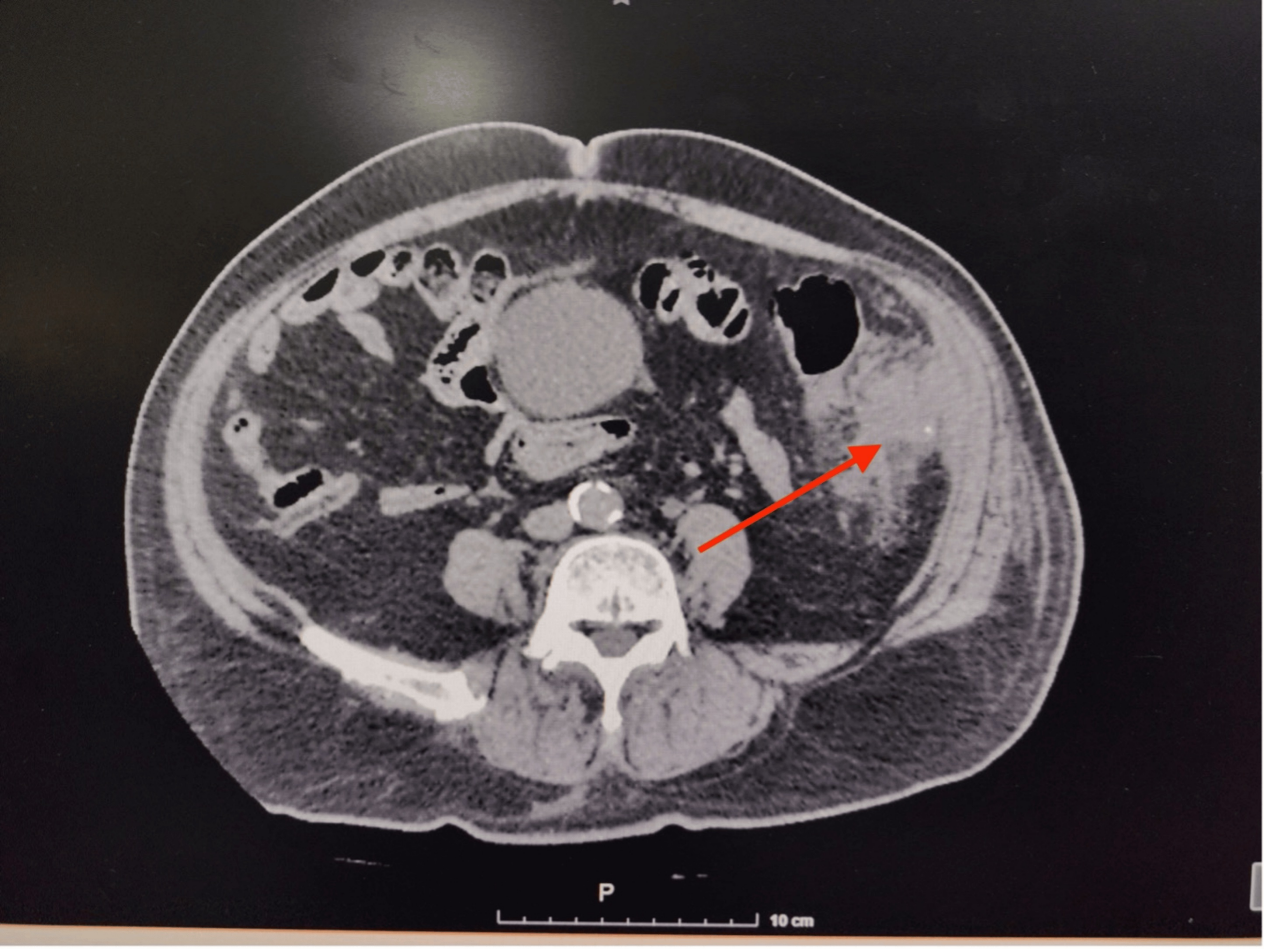
CT scan revealed a mass adjacent to the descending colon with peripheral enhancement dependent on the colonic wall, extending into the muscular layer, suggestive of an abscess, as well as a small radio-opaque object in the middle of the phlegmon, supportive of a foreign body

**Figure 2:**
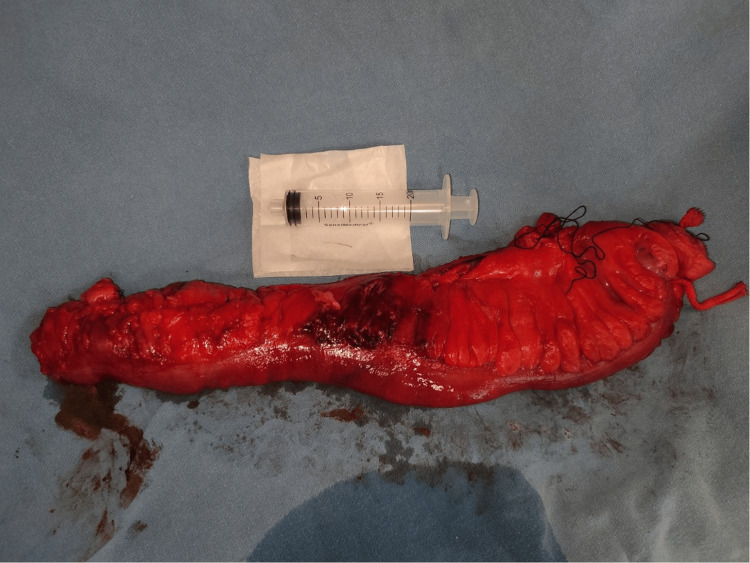
Left hemicolectomy surgical piece

**Figure 3:**
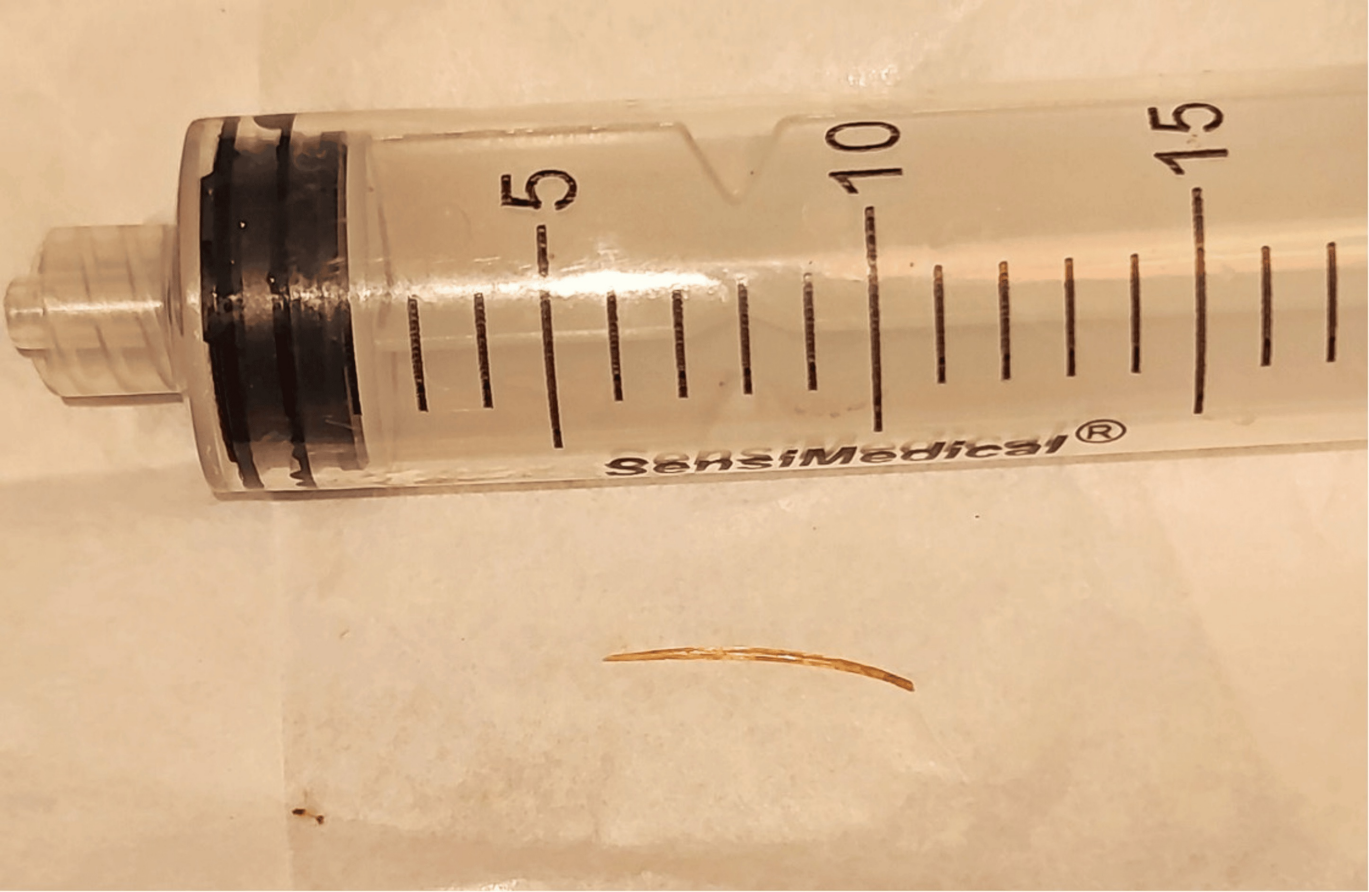
Fish bone after surgical resection

An 80-year-old male patient with a history of diabetes mellitus and hypertension. No prior surgical history nor screening colonoscopy. He was referred to our service for a 12-day history of lower left quadrant abdominal pain, progressively worsening to become intolerable, prompting emergency admission. On presentation, the patient was stable but exhibited abdominal distension and tenderness on palpation of the left hemiabdomen, without signs of peritoneal irritation. Laboratory tests showed leukocytosis of 20,800/μL (4,000-11,000), neutrophils at 65.1% (40%-70%) of total leukocytes, and a serum creatinine of 1.0 mg/dL (0.6-1.2) Contrast-enhanced abdominal and pelvic CT scan revealed a mass adjacent to the descending colon with peripheral enhancement dependent on the colonic wall, extending into the muscular layer, suggestive of an abscess, as well as a small radio-opaque object in the middle of the phlegmon supportive of a foreign body (Figure 1). There was adjacent fat stranding and free fluid in the left iliac fossa. The patient underwent exploratory laparotomy, revealing a colonic perforation at the sigmoid colon with firm adhesions to the abdominal wall. A sharpened foreign body approximately 2 cm in length was found at the adhesion site, likely the cause of perforation. No diverticula were identified in the colon. A left hemicolectomy with transverse colon terminal colostomy was performed (Figures 2-3). Postoperative recovery was uneventful under antibiotic therapy with meropenem and metabolic management. The patient was discharged home on postoperative day 5 with clinical improvement. The histopathology report stated chronic inflammation with no evidence of malignancy.

## Discussion

Foreign body ingestion is a common global problem, with an estimated incidence of 120 per million, and is responsible for nearly 1,500 deaths annually. Endoscopic retrieval is required in 10-20% of cases, and approximately 1% develop intestinal perforation. The most significant risk factor for fish bone ingestion is the use of dentures. Additional risk factors include advanced age, childhood, rapid eating, psychiatric conditions, or mental health alterations [1].

Presentations vary widely, including abdominal abscesses, fistulas (colorectal, colovesical, or enterovesical), pseudotumor formations, gastrointestinal bleeding, or perforation. Perforation can occur anywhere along the gastrointestinal tract, but is most common in angulated regions such as the ileocecal or rectosigmoid junctions [4].

The ileum accounts for approximately 39% of perforations, followed by the jejunum at 27%, with colonic perforations in just about 9% of cases. The most common symptom is abdominal pain, present in approximately 95% of patients, followed by fever in 81%, caused by localized peritonitis in 39% or generalized peritonitis in 27% [5].

Computed tomography (CT) plays a crucial role, with 100% sensitivity and specificity, providing information on foreign body localization, abscess presence, or perforation signs such as free air and intra-abdominal fluid.

Surgical management is preferred in cases of peritonitis, abscess, severe inflammation, or significant bleeding. Segmental resection remains the treatment of choice. Non-surgical approaches may be suitable depending on perforation size and location, time to diagnosis, patient stability, and degree of abdominal sepsis [6].

In elderly patients, intestinal perforation by fish bones can mimic colon cancer [7].

In our case, the foreign body was not detected specifically on CT, and cancer was a differential diagnosis, especially due to the absence of colonic diverticula in imaging studies. Histology confirmed an inflammatory process at the perforation site without evidence of malignant cells.

## Conclusions

Intestinal perforation by a fish bone in the colon is a rare complication that poses diagnostic challenges. It requires a high index of suspicion from surgeons or emergency physicians. In patients with risk factors, such perforations can resemble tumors with abscess formation or perforation secondary to malignancy.
